# Optimized Platelet-Rich Plasma Preparations for a Consistently High Platelet Capture Rate, Bioformulation Flexibility, and Red Blood Cell Reduction Using a Single-Spin Device

**DOI:** 10.3390/bioengineering13070780

**Published:** 2026-07-07

**Authors:** Walter Sussman, Jane Fitzpatrick, Ariana DeMers, Peter A. Everts

**Affiliations:** 1 Boston Sports & Biologics, Wellesley, MA 02481, USA; wsussman@gmail.com; 2 PM&R, Medical Center, Tufts University, Boston, MA 02111, USA; 3 Faculty of Medicine, Dentistry and Health Sciences, University of Melbourne, Parkville, VIC 3010, Australia; jane.fitzpatrick@unimelb.edu.au; 4 Joint Vitality Institute, Sonora, CA 95370, USA; arianademers@gmail.com; 5 Medical School (GBCS), The University of Queensland, Brisbane, QLD 4006, Australia; 6 Medical School, Max Planck University Center (UniMAX), OrthoRegen, Indaiatuba 13343-060, SP, Brazil

**Keywords:** orthobiology, centrifugal density separation, platelet-rich plasma, full buffy coat-poor PRP, leukocyte-rich PRP, bioformulations, platelet concentration, platelet capture rate, mean platelet volume, flexibility

## Abstract

The preparation of platelet-rich plasma (PRP) requires precise density-based centrifugation of anticoagulated whole blood to achieve an optimal hematologic bioformulation while enhancing platelet recovery efficiency. Commercial PRP systems exhibit substantial heterogeneity in processing architecture, with variable platelet yields and inconsistent cellular composition profiles. In this clinical PRP device evaluation, 70 sequential samples sourced from two independent clinical facilities were used to evaluate the performance characteristics of the XCELL 60 mL single-spin centrifugation platform. Two different PRP preparations were consistently prepared as per physician preferences: PRP-1 and PRP-2. The main differences between these two preparations were the concentration of leukocytes and reduction in red blood cells. The system was evaluated based on critical PRP performance metrics. The results demonstrated the following: (1) A consistent 8-fold increase in platelet concentration relative to baseline whole blood was achieved. (2) The average platelet capture rate (PCR) was 83%. (3) The total available platelets (TAPs) in the PRP specimen produced from both groups combined were 10.8 ± 2595 billion platelets within a final product volume of 6 mL. (4) Hematocrit values were reduced to <2–6% across sites (reduction of 94% and 84% in RBCs, respectively). Finally, (5) a customizable leukocyte content (20.9–25.4 × 10^9^/L) was achieved without comprising platelet yield. This single-spin centrifugation architecture achieved performance parity with historically preferred double-spin systems while reducing the processing time and number of preparation steps. Engineering analysis established that high-precision platelet recovery and bioformulation control are achievable through optimized single-spin centrifugal design, enabling standardized therapeutic dosing for autologous regenerative medicine applications.

## 1. Introduction

Platelet-rich plasma (PRP) is an autologous biologic defined by a platelet concentration (PLTc) exceeding baseline whole-blood levels, and it is prepared to deliver supraphysiologic platelet-derived cytokines and growth factors to modulate inflammation and stimulate tissue repair [1,2]. A total platelet dose of approximately 1 billion platelets in a PRP preparation is increasingly recognized as a clinically relevant parameter for characterizing platelet delivery, although it is not a universally accepted regulatory definition of PRP [3]. Foundationally, centrifugation-based processing concentrates platelets and associated α-granule factors (for example, platelet-derived growth factor (PDGF), transforming growth factor-beta (TGF-β), and vascular endothelial growth factor (VEGF)), though protocols vary in anticoagulants, forces, and times, contributing to heterogeneity in the final product composition [4].

The use of PRP has expanded across musculoskeletal indications—including osteoarthritis, tendinopathies, and ligament injuries—and it now constitutes a widely deployed orthobiological agent in orthopedics and sports medicine, paralleling a marked rise in clinical studies and reviews over the past two decades [5,6]. Its clinical effectiveness is inconsistent, with randomized trials and meta-analyses reporting benefits in pain and function in some settings and null effects in others, a pattern largely attributed to variability in PRP bioformulations—platelet dose, leukocyte content, and red blood cell (RBC) contamination—across commercial systems and protocols [7,8,9]. Comparative analyses have shown more than tenfold differences in platelet recovery and leukocyte/RBC carryover between devices, with some producing leukocyte-rich PRP (LR-PRP) and others producing leukocyte-poor PRP (LP-PRP); these compositional differences likely underpin divergent biologic responses and outcomes [8,10]. Moreover, patient factors (e.g., baseline platelet count and age) and operator/process variables further modulate yield and reproducibility, reinforcing the need to report absolute platelet dose, recovery efficiency, purity, and activation status (for example, DEPA classification) to enable interpretation and standardization [11,12,13].

Whole-blood cellular isolation and concentration requires gravitational separation of hematologic components based on density differences. Currently, two primary centrifugation architectures dominate commercial systems: double-spin protocols, which utilize sequential centrifugation cycles to achieve progressive plasma/platelet/erythrocyte layer separation [14], and single-spin protocols, which employ a single centrifugal event to achieve simultaneous multi-phase gravitational stratification. Historically, double-spin systems were presumed necessary to achieve optimal blood phase separation for higher platelet recovery, albeit at the cost of time and complexity; early comparative studies noted greater concentrations but potential platelet activation and morphology changes versus single-spin approaches [15,16].

However, single-spin systems theoretically offer substantial engineering advantages: a reduced processing time, a simplified protocol architecture, minimized cellular mechanical trauma through fewer centrifugal cycles, and reduced contamination risk through fewer tube transfer operations [17,18]. Traditionally, many single-spin test tube-like kits underperform in capture, but modern single-spin methods can achieve ≥ 50% platelet yield with moderate variability and enable LP-PRP in some configurations, suggesting that efficiency is feasible without a double-spin process in select systems [19].

The evaluated PRP system (APEX Biologix, Clearwater, FL, USA) is designed to achieve high platelet capture rates through a single-spin process and can produce distinct PRP bioformulations, including LP-PRP and LR-PRP. However, peer-reviewed performance data remain limited, underscoring a current evidence gap specific to this platform [20]. The studied PRP system represents a bioengineering innovation designed to overcome traditional single-spin performance limitations through optimized centrifugal chamber geometry; proprietary separation algorithms; and a controlled PRP extraction interface, namely, the Benchtop Processing Station (APEX Biologix, Clearwater, FL, USA) (BPS)). The engineering objective of the device is to achieve platelet capture efficiency and platelet concentration ratios equivalent to or exceeding those of double-spin systems while maintaining the operational advantages of a single-spin architecture (reduced time and a simplified protocol), with the ability to produce different PRP bioformulations.

This two-center study evaluates a 60 mL PRP system by quantifying key parameters in the preparation of two different PRP bioformulations. By utilizing a variety of study parameters, we can compare how the system performs across both bioformulations within the same device. The study’s findings are contextualized within the broader framework of contemporary literature, which emphasizes the critical need for standardized reporting of TAPs and formulation characteristics in PRP research. This standardization is essential to address the variability in PRP preparation methods and reporting practices, which has historically hindered cross-study comparability and the ability to draw consistent conclusions about the efficacy of PRP therapies. By providing detailed and reproducible data on platelet and cellular metrics, this study contributes to the growing body of evidence that supports the application of PRP in clinical settings [21,22]. Furthermore, the emphasis on TAPs as a key outcome measure aligns with emerging consensus in the field that quantifying the absolute platelet yield is a more meaningful indicator of PRP quality and potential therapeutic impact than simply reporting relative platelet concentrations [23].

This work not only provides valuable insights into the performance of the specific 60 mL PRP system but also reinforces the importance of adopting standardized methodologies and reporting frameworks to enhance the reliability and interpretability of PRP studies. Such efforts are crucial for advancing the field and facilitating the evidence-based integration of PRP therapies into routine clinical practices where PRP is increasingly utilized to support tissue healing and repair.

## 2. Materials and Methods

### 2.1. Study Design Ethical Approval

Following institutional review board approval (BRANY, DB2024-001) of this prospective, multicenter study, data were collected at two outpatient physiatry and orthopedic clinics (April–August 2025) that use two different PRP formulations, one prepared via buffy coat collection (PRP-1) and one prepared via full buffy coat collection with adding 0.5 mL of the RBC layer (PRP-2), to treat a variety of musculoskeletal pathologies, according to the physicians’ preferences. Reporting standards recommended by the Minimum Information for Studies Evaluating Biologics in Orthopaedics guidelines were used to ensure reproducibility of the data [24].

This study evaluates a 60 mL PRP system by quantifying several key parameters essential for understanding the performance and characteristics of PRP preparations. These parameters include the following:

• Platelet concentration, which provides a direct measure of the platelet density in the final product;
• Platelet concentration fold increase relative to baseline, which indicates the degree of platelet enrichment achieved through the processing method compared to the initial blood sample;
• Platelet capture rate efficiency, a metric that reflects how effectively platelets are recovered during the centrifugation process;
• Total available platelets (TAPs), a critical measure of the absolute number of platelets available for therapeutic use in the PRP formulation;
• Cellular composition, which encompasses the total number of leukocytes, their differentiation profiles (e.g., monocytes, lymphocytes, and neutrophils), and the presence of red blood cells (RBCs), all of which can influence the biologic activity and clinical applicability of the PRP.

### 2.2. PRP Preparation

Whole-blood and PRP samples were obtained and analyzed on the same day to reflect routine clinical practice. Using clinical practice guidelines for phlebotomy, a total of 54 mL of whole blood was collected and combined with an anticoagulant solution [25]. Directly after the blood draw, the syringe was agitated for proper anticoagulation mixing, prior to transfer to the XCELL single-spin PRP device (Apex Biologix, Clearwater, FL, USA).

**Figure 1:**
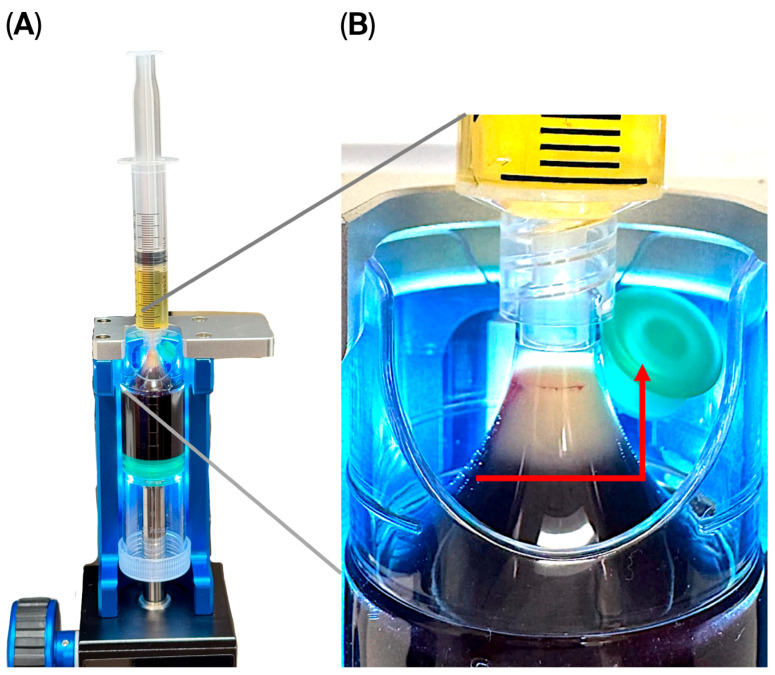
In (**A**), the entire BPS is shown with the PRP device after centrifugation and prior to PRP extraction. In (**B**), the buffy coat stratum after centrifugation is displayed. Following the removal of the PPP, the buffy coat stratum is clearly concentrated in the cone of the PRP device (between the red lines), prior to PRP extraction.

A Drucker Boost 4+ Flex centrifuge (Drucker Diagnostics, Port Mathilda, PA, USA) was used for whole-blood centrifugation at 3500 rpm for 10 min (2300× *g* Relative Centrifugal Force, RCF) to stratify plasma, buffy coat, and red blood cells, consistent with single-spin methodologies that target ≥ 50% platelet yield with tunable leukocyte carryover [20]. The proprietary BPS facilitated visualization of blood layers and controlled PRP extraction, capturing either PRP-1 or PRP-2, as shown in Figure 1.

#### 2.2.1. PRP-1 Protocol

**Figure 2:**
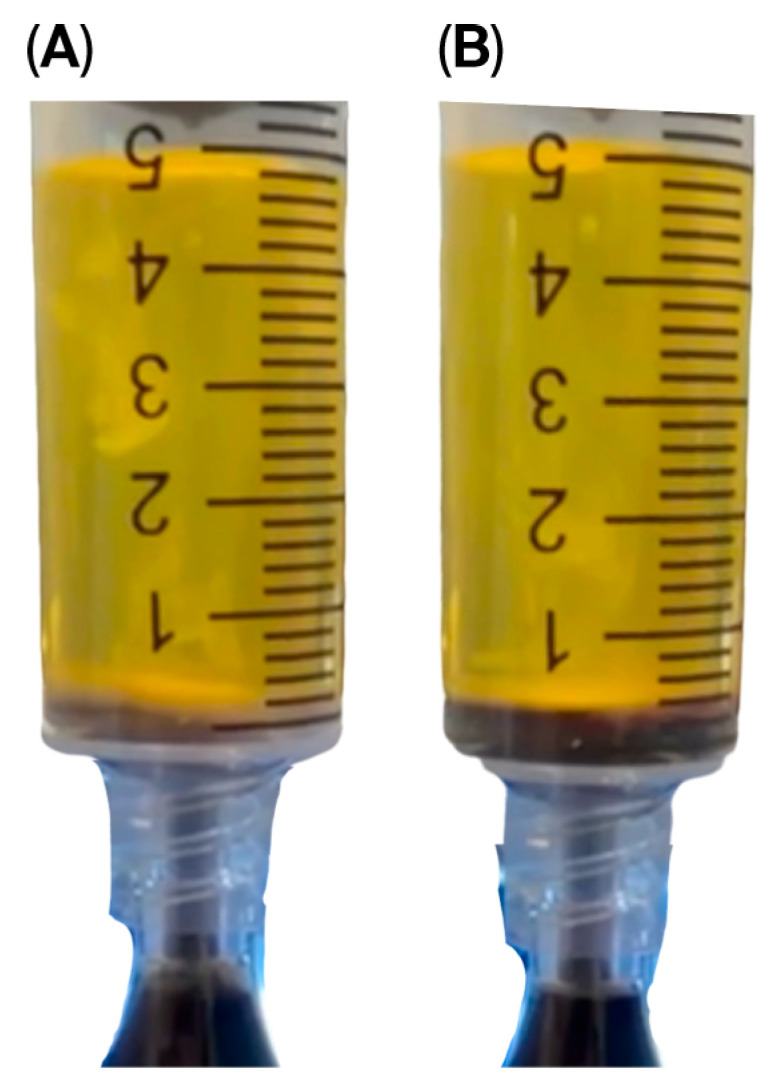
PRP bioformulation extractions. As shown in (**A**), by using the BPS in a controlled manner, PRP-1 was obtained by extracting the buffy coat, with a flair of red-colored cells entering the PRP syringe. As shown in (**B**), the buffy coat was captured with the addition of 0.5 mL of the RBC layer, representing PRP-2, before agitation to consolidate the specimen.

PRP-1 was obtained by first aspirating the platelet-poor plasma using the BPS and a 60 mL syringe attached to the port of the concentrating device until the buffy coat reached the 6 mL mark. The 60 mL PPP collection syringe was then replaced by a 10 mL syringe, and PRP-1 was collected with only a “flair” of red-colored cells entering the PRP syringe to minimize the presence of leukocytes (Figure 2A).

#### 2.2.2. PRP-2 Protocol

The PRP-2 protocol captured the buffy coat and went approximately 0.5 mL deeper into the RBC layer, with the aim of capturing a higher leukocyte content (Figure 2B).

### 2.3. Laboratory Analysis

Baseline whole blood and the resultant PRP were analyzed on a Horiba Micros 60 hematology analyzer (Horiba ABX Micros Series Hematology Analyzer, Montpellier, France) to quantify platelets, leukocytes, and red blood cells. The analyzers were calibrated prior to the study according to the company guidelines.

The calculated metrics included the platelet concentration factor (fold increase over the baseline), the TAPs in the PRP volume, the PCR (percentage of platelets captured from the pre-processed whole blood), and the formula for RBC depletion % are shown in below formulations, Equation, as described by Everts et al. [26]. Additionally, the mean platelet volume (MPV) in the whole blood and PRP of all patients was determined; this laboratory parameter was measured as part of the complete blood count (CBC) analysis, and it represents the average size of platelets and, thus, the regenerative potential of the PRP, measured in femtoliters (fL) [27].

Formulations used to calculate the PRP metrics [26]:

Fold increase = PLTc_PRP_/PLTc_WB_

Capture rate % = (PLTc_PRP_ × V_PRP_ )/(PLTc_WB_ × V_WB_) × 100 

Total PLT_PRP_ = (PLTc_PRP_ × V_PRP_) 

RBC depletion % = [1 − (RBC_PRP_/RBC_WB_)] × 100

Abbreviations: PRP: platelet-rich plasma; WB: whole blood; PLTcPRP: platelet concentration of PRP; VPRP: volume of PRP; PLTcWB: platelet concentration in WB; VWB: volume of WB; RBCPRP: red blood cell concentration in PRP; RBCWB: red blood cell concentration in WB.

### 2.4. Statistical Analysis

All statistical computations (descriptive statistics, Pearson’s product moment correlations, *p*-values, 95% confidence intervals, linear regressions, and graphics) were performed using SAS statistical software (SAS Institute Inc., Cary, NC, USA; version 9.4) or R version 4.5.3 with RStudio 2026.01.1+403 “Apple Blossom” Release. The *t*-test was used for the hypothesis testing of means and correlations. All hypothesis testing was two-tailed. A *p*-value of 0.05 or less was considered significant. Satterthwaite adjustment was used for unequal variances.

## 3. Results

### 3.1. Patient Demographics

A total of 70 participants (with a mean age of 54.0 years, SD of 15.0), of whom 35 were male, underwent venipuncture with a 60 mL syringe preloaded with 6 mL of Anticoagulant Citrate Dextrose Solution A (ACD-A); 54 mL of whole blood was aspirated to prepare PRP bioformulations for the treatment of a variety of musculoskeletal pathologies. CBC analysis revealed that there were no statistical differences between the two PRP groups in terms of the baseline leukocyte count. However, PRP-1-treated patients had a statistically higher baseline platelet concentration than PRP-2-treated patients (*p* = 0.0002). Additionally, these patients had a significantly higher baseline MPV (*p* < 0.0001).

Overall, the PRP system demonstrated consistent performance among the two centers in producing PRP-1 and PRP-2 treatment volumes, with 6.0 ± 0.2 and 6.5 ± 0.4 mL, respectively (NS). The PRP device performance data are shown in Table 1.

### 3.2. Differences in Cellular Composition Between PRP-1 and PRP-2 Preparations

The operational flexibility of the PRP device facilitated the extraction of either PRP-1 or PRP-2 bioformulations with distinct differences in cellular contents (Table 2).

PRP-1 yielded a higher platelet concentration (2031 vs. 1533 × 10^3^/µL) while exhibiting greater purity, with markedly reduced red blood cell contamination (0.24 vs. 0.69 × 10^9^/µL), lower hematocrit values (1.9% vs. 6.1%), and fewer neutrophils (3.4 vs. 7.5 × 10^3^/µL). PRP-2 furnished a higher total leukocyte count (25.4 vs. 20.9 × 10^3^/µL), predominantly due to roughly double the neutrophil count, along with greater erythrocyte contamination but a lower platelet yield. Notably, WB parameters were not significantly different between the groups, indicating that the observed differences stemmed from the preparation method rather than donor variation.

#### 3.2.1. Platelet Concentrations, TAPs, and MPV

The TAPs in the PRP-1 preparation (12,199 × 10^9^ ± 2175) were significantly higher than in the PRP-2 preparation (9898 × 10^9^ ± 2134) because the baseline platelet concentration was higher in this group (*p* < 0.001). Consequently, this contributed to a significantly higher increase in the platelet concentration factor above the baseline (*p* = 0.0188) compared to PRP-2. Nevertheless, the device PCR performance was identical for both bioformulations, at 83%.

**Figure 3:**
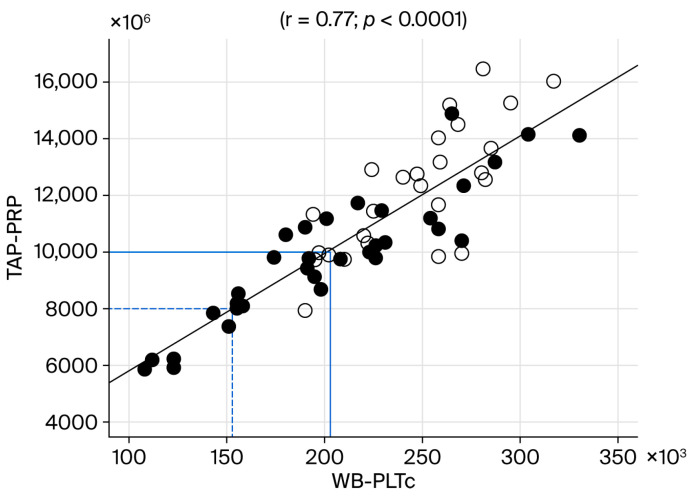
Correlation between WB = PLTc and TAP-PRP. The TAPs in both PRP bioformulations are highly significantly correlated (r = 0.77) with the WB-PLTc volume (*p* < 0.001). Black dots represent PRP-2 preparations.

Pearson’s correlation analysis of the whole-blood platelet concentration and all PRP platelet concentrations revealed a highly significant correlation (*p* < 0.0001). In patients with a WB-PLTc of 208 × 10^3^/µL, the device was able to concentrate ≥ 10 billion TAPs in any PRP bioformulation, as shown in Figure 3 (solid blue lines). In patients with a WB-PLTc of >161 × 10^3^/µL, the system yielded ≥ 8 billion TAPs (dashed blue lines).

The two PRP bioformulations were compared in terms of platelet size, measured as the MPV. It was found that the platelet size significantly increased only in the PRP-2 preparations, where it was 11.1% greater (*p* = 0.0003). Furthermore, the increase in larger PRP platelets was significantly correlated with the MPV value (r = −0.54; *p* < 0.0001), as shown inFigure 4. In PRP-1, the MPV was not correlated with the platelet size (r = −0.3; *p* = 0.11).

#### 3.2.2. Leukocytes

As PRP-1 was captured with fewer inflammatory cells, it showed a significantly lower total leukocyte content (*p* = 0.0081) than PRP-2 (20.9 ± 5.7 and 25.4 ± 7.8 × 10^3^/µL, respectively). The PRP-2 preparation was designed to contain a higher leukocyte content than the PRP-1 preparation (*p* = 0.0081). Accordingly, the monocyte concentration was highly significantly increased in this bioformulation, reaching 2.88 ± 1.16 × 10^3^/µL compared to 2.20 ± 0.54 × 10^3^/µL in PRP-1 (*p* = 0.0018). A correlation analysis of the monocyte and neutrophil concentrations in the two PRP bioformulations showed no association with the PRP MPV values (r = 0.338 and 0.007 for monocytes in PRP-1 and PRP-2, respectively; r = 0.0095 and 0.038 for neutrophils in PRP-1 and PRP-2, respectively).

#### 3.2.3. Red Blood Cells

The average RBC concentration of PRP-1 was 0.24 × 10^6^/µL, corresponding to a reduction of 94%. Despite the fact that the PRP-2 bioformulation captured the buffy coat, plus an additional 0.5 mL of the RBC layer, the mean RBC concentration was 0.69 × 10^6^/µL, corresponding to a reduction of 84% in this preparation compared to the whole-blood baseline values.

## 4. Discussion

This multicenter evaluation demonstrated consistent and high PCR efficiency, with the two sites achieving identical capture percentages (83%) for PRP-1 and PRP-2 formulations. A significant difference in the TAPs was noted between the two bioformulations, which was attributed to the significantly higher baseline platelet concentrations in the PRP-2 cohort (*p* = 0.0002). The consistent capture percentages indicate that the baseline PLTc is not correlated with the PCR. Overall, the study population achieved clinically important high TAP numbers, with a combined average platelet dose of 10.8 billion platelets in a 6.3 mL PRP sample. Notably, the device was able to concentrate ≥10 billion TAPs when the baseline WB PLTc was >208 × 10^3^/µL in any PRP bioformulation and ≥8 billion TAPs when the WB-PLTc was >161 × 10^3^/µL. These TAP numbers underscore a significant advancement in achieving standardized therapeutic dosing, as mentioned in the literature [28,29,30].

PRP has emerged as a significant orthobiological agent within orthopedic and sports medicine fields [31,32]. The therapeutic rationale for PRP administration rests on its capacity to deliver bioactive growth factors and cytokines that facilitate tissue regeneration and repair processes [33,34]. Despite this theoretical foundation and growing clinical adoption, substantial heterogeneity in PRP preparation methodologies presents a fundamental impediment to achieving consistent clinical efficacy and standardized treatment protocols. The variability inherent in commercially available PRP devices extends beyond differences in preparation techniques alone, as these devices demonstrate significant disparity in their architecture, centrifugation parameters, and processing algorithms, culminating in inconsistent platelet recovery rates and compositionally distinct cellular formulations. Notably, comparative analyses of device performance have documented platelet capture efficiency variations exceeding tenfold differences between systems, underscoring the magnitude of technical heterogeneity in current clinical practice [10]. Furthermore, while PRP bioformulations can be categorized into discrete classifications according to established nomenclature systems [35,36], the technical capacity to produce multiple PRP formulations remains limited to a restricted subset of available devices, substantially constraining opportunities for therapeutic personalization and individualized treatment optimization. Consequently, the cumulative effect of this heterogeneity in device performance and formulation capability likely accounts for the conflicting clinical outcomes documented in the literature, wherein some investigations demonstrate therapeutic efficacy while others fail to establish statistically significant clinical benefits.

A key feature of the used PRP system is its ability to generate bioformulations with distinct leukocyte profiles while maintaining identical PCRs. As shown in Table 2, the addition of approximately 0.4 mL of red-colored cells increased the leukocyte content as the entire buffy stratum was collected, including a higher number of cells with a similar density to RBCs. PRP-1 exhibited a significantly lower total leukocyte content than the PRP-2 bioformulation (*p* = 0.0081), whereas PBC-PRP, which retained a higher leukocyte content through complete buffy coat capture, demonstrated an increased monocyte concentration compared to PRP-1, potentially facilitating a more rapid transition from macrophage type 1 to macrophage type 2 and pro-repair signaling [37]. The flexibility in creating different PRP bioformulations represents a notable therapeutic advantage, enabling the independent modulation of leukocyte content without compromising platelet capture efficiency. In contrast to systems exhibiting inverse relationships between leukocyte content and platelet yield [38], this PRP platform enables clinicians to customize PRP bioformulations in order to address specific biologic requirements. The optimal leukocyte concentration in PRP is not universally beneficial or harmful and should be interpreted cautiously, as it depends on the clinical indication with no firm consensus. Clinicians should view recommendations as provisional and tailor the leukocyte content to the tissue and condition. The role of leukocytes in PRP is debated and requires high-quality, standardized research for definitive guidance.

The presence of red cells in PRP has been poorly studied in the literature; however, there is a general consensus that PRP preparation protocols should aim to reduce the RBC content [30]. Unlike platelets and leukocytes, RBCs do not seem to contribute to tissue regeneration and, thus, appear to be redundant; however, they may cause harm to musculoskeletal tissues [39]. Conversely, recent evidence describes the potential immunomodulatory, angiogenic, and regenerative properties of trace amounts of RBCs [40]. As demonstrated in the PRP preparations, this system achieves significant erythrocyte depletion, minimizing the presence of red blood cells that could otherwise compromise the quality and functionality of the final PRP product.

The MPV, quantified through hematological analysis via CBC methodology, constitutes a critical determinant for prognosticating platelet functionality and regenerative capacity [41]. According to the observations of Leader et al., platelets exhibiting elevated MPV values, which serve as indicators of morphologically larger and biologically younger cellular populations, demonstrate significantly enhanced granular compartmentalization, encompassing both alpha and dense granule populations, in conjunction with augmented adhesion molecule expression [41,42]. These enhanced cellular characteristics consequently provide such platelets with a substantially elevated capacity for orchestrating tissue repair mechanisms and modulating inflammatory responses [43]. Given these superior functional attributes inherent in high-MPV platelet populations, strategic PRP formulation selection warrants deliberate calibration to maximize these regenerative properties. This bioanalytical rationale substantiates the selective application of PRP-2 formulations when the synergistic interactions between concentrated platelet populations and elevated leukocyte concentrations may potentiate therapeutic efficacy through their complementary enzymatic and angiogenic mechanisms or, alternatively, the procurement of the complete buffy coat layer to capitalize upon elevated monocyte concentrations in tandem with platelet preparations demonstrating higher MPV characteristics [5,31]. Through such formulation-specific implementation strategies informed by hematological parameters, clinicians may optimize therapeutic outcomes by leveraging quantifiable markers of platelet quality and biological potential.

In this study, it was found that the platelet size in PRP-2 preparations was 11.1% greater than that in PRP-1 (*p* = 0.0003), indicating enhanced functional significance. This difference in platelet size is attributed to variations in the PRP extraction protocols for each bioformulation. Specifically, PRP-2 captures the buffy coat plus an additional 0.5 mL of the RBC layer, which includes a higher concentration of larger platelets. The consistent production of larger platelets in PRP-2 suggests that this preparation may offer greater regenerative potential. Larger platelets are associated with a higher availability of bioactive molecules, including growth factors such as PDGF, VEGF, and TGF-TGF-β, as well as cytokines that play critical roles in tissue repair and immune modulation, including platelet-derived exosomes [44,45]. Additionally, the increased surface area and granular content of larger platelets may enhance their ability to activate platelets and thus release increased concentrations of bioactive substances [46].

Notably, a key finding was a significant inverse correlation between the TAPs and MPV in PRP-2 preparations (r = −0.54, *p* < 0.0001), indicating that the TAPs are associated with larger platelets, offering both high platelet dosing potential and more active platelets in patients in whom PRP-2 is employed. In contrast, PRP-1 showed no significant correlation between the TAPs and MPV, suggesting that the leukocyte minimization protocol disrupts the natural size–quantity relationship, resulting in more uniform smaller platelet sizes [47]. This implies that PRP-2 may provide a dual advantage of higher doses and larger platelets, while PRP-1 standardizes platelet size across patients [47,48].

The data show that the single-spin PRP device used in this study can consistently produce distinct PRP bioformulations without compromising the yield of TAPs, which is attributed to the device’s high PCR. This indicates that the device is highly efficient in consistently isolating and concentrating platelets while maintaining the cellular integrity of any PRP bioformulation and composition. This versatility within a single device architecture provides clinicians with the flexibility to adapt PRP treatments to the specific clinical context and desired therapeutic outcomes.

## 5. Conclusions and Perspectives

This comprehensive analysis demonstrates that the XCELL single-spin centrifugation platform achieves a critical objective in PRP preparation by reliably and consistently producing high-quality PRP bioformulations that meet key clinical and biophysical standards. Specifically, the platform ensures consistently high PCRs, enabling reproducible therapeutic outcomes, along with customizable leukocyte profiles that allow clinicians to tailor PRP formulations to specific biologic requirements, such as the modulation of inflammatory or regenerative responses. Moreover, the system effectively depletes erythrocytes to minimize the presence of blood cells that might compromise the final PRP content and quality.

A key finding of this analysis is the establishment of the baseline platelet concentration as the primary determinant of the TAPs achievable in the final formulation. This insight underscores the importance of patient-specific platelet counts in predicting PRP yield and highlights the need for personalized approaches to PRP therapy. Furthermore, this study demonstrates that PRP-2 formulations consistently produce significantly larger platelets than other formulations, a feature that is observed independent of neutrophil enrichment. This suggests that the biophysical properties of platelets, such as size and associated granular content, are influenced by the extraction protocol rather than solely by the leukocyte content, providing valuable mechanistic insights into how centrifugation and bioformulation processes impact platelet characteristics to optimize the regenerative potential and maximize the probability of achieving clinically significant benefits in tissue repair applications.

## Figures and Tables

**Figure 4:**
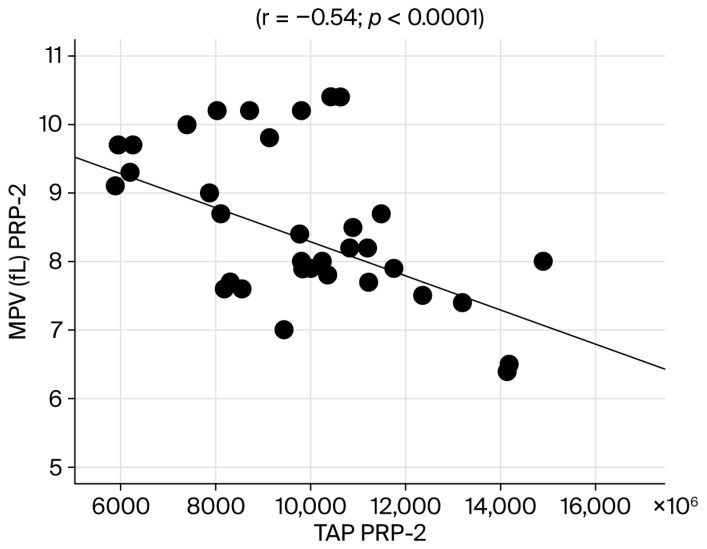
Correlation between the MPV and TAPs in PRP-2. The MPV is significantly negatively correlated with the TAPs in PRP-2 (r = −0.54 and *p* < 0.001).

**Table 1:** PRP device performance data for PRP-1 and PRP-2 preparations, expressed as mean and SD.

Metric	PRP-1 *N* = 26 (Mean ± SD)	PRP-2 *N* = 44 (Mean ± SD)	*p*-Value
X-fold increase PLTc	8.27 ± 1.05	7.68 ± 0.80	*p* = 0.018
PCR, %	83 ± 9	83 + 9	NS
TAPs, (10^9^)	12,199 ± 2175	9898 ± 2134	*p* < 0.001
RBC depletion, %	94 ± 2	84 ± 1	*p* < 0.001

Abbreviations: PLTc: platelet concentration; PRP: platelet-rich plasma; PRP-1: buffy coat plus flair of red cells; PRP-2: buffy coat plus 5 mL of RBC layer; PCR: platelet capture rate; TAPs: total available platelets; RBC: red blood cell; SD: standard deviation; NS: not significant.

**Table 2:** CBC data for baseline whole blood and PRP bioformulation. Data are expressed as mean with SD and *p*-value.

CBC Parameters	PRP-1 *N* = 26 (Mean ± SD)	PRP-2 *N* = 44 (Mean ± SD)	*p*-Value
WB PLTc (10^3^/µL)	246 ± 35	203 ± 54	*p* = 0.0002
PRP PLTc (10^3^/µL)	2031 ± 388	1533 ± 342	*p* < 0.0001
WB WBCs (10^3^/µL)	5.22 ± 1.2	5.62 ± 1.5	NS
PRP WBCs (10^3^/µL)	20.9 ± 5.8	25.4 ± 7.9	*p* = 0.0081
WB Mon (10^3^/µL)	0.44 ± 0.7	0.38 ± 0.2	NS
PRP Mon (10^3^/µL)	2.2 ± 0.6	2.9 ± 1.2	*p* = 0.0018
WB Neu (10^3^/µL)	3.2 ± 0.9	3.4 ± 1.2	NS
PRP Neu (10^3^/µL)	3.4 ± 2.5	7.5 ± 5.3	*p* < 0.001
WB RBCs (10^9^/µL)	4.3 ± 0.5	4.3 ± 0.4	NS
PRP RBCs (10^9^/µL)	0.24 ± 0.09	0.69 ± 0.49	*p* < 0.001
WB HT, %	40.9 ± 4.8	41.2 ± 3.7	NS
PRP HT, %	1.9 ± 0.8	6.1 ± 4.7	*p* < 0.001
WB MPV, fL	6.77 ± 0.60	7.59 ± 0.97	*p* < 0.001
PRP MPV, fL	7.49 ± 0.73	8.32 ± 1.07	*p* = 0.0003

Abbreviations: WB: whole blood; PLTc: platelet concentration; PRP: platelet-rich plasma; WBCs: white blood cells; Mon: monocytes; Neu: neutrophils; RBC: red blood cell; HT: hematocrit; MPV: mean platelet volume; SD: standard deviation; NS: not significant.

## Data Availability

Data are not available due to privacy restrictions

## Abbreviations

The following abbreviations are used in this manuscript:

ACD-A	Anticoagulant Citrate Dextrose Solution A
BPS	Benchtop Processing Station
CBC	Complete Blood Count
fL	Femtoliter
LR-PRP	Leukocyte-Rich PRP
LP-PRP	Leukocyte-Poor PRP
Mon	Monocyte
MPV	Mean Platelet Volume
Neu	Neutrophil
NS	Not Significant
PCR	Platelet Capture Rate
PLTc	Platelet Concentration
PRP	Platelet-Rich Plasma
P	*p*-value
PDGF	Platelet-Derived Growth Factor
RBC	Red Blood Cell
RCF	Relative Centrifugal Force
SD	Standard Deviation
TGF-β	Transforming Growth Factor-Beta
TAPs	Total Available Platelets
VEGF	Vascular endothelial growth factor
WB	Whole Blood
WBCs	White Blood Cells
